# Transcriptome-Wide Association Studies and Integration Analysis of mRNA Expression Profiles Identify Candidate Genes and Pathways Associated With Ankylosing Spondylitis

**DOI:** 10.3389/fimmu.2022.814303

**Published:** 2022-05-10

**Authors:** Ruoyang Feng, Mengnan Lu, Lin Liu, Ke Xu, Peng Xu

**Affiliations:** ^1^ Department of Joint Surgery, HongHui Hospital, Xian Jiaotong University, Xi’an, China; ^2^ Department of Pediatrics, The Second Affiliated Hospital of Xi’an Jiaotong University, Xi’an, China

**Keywords:** ankylosing spondylitis, genome-wide association study (GWAS), transcriptome-wide association study (TWAS), mRNA expression profile, susceptibility genes

## Abstract

This study aimed to identify susceptibility genes and pathways associated with ankylosing spondylitis (AS) by integrating whole transcriptome-wide association study (TWAS) analysis and mRNA expression profiling data. AS genome-wide association study (GWAS) summary data from the large GWAS database were used. This included data of 1265 AS patients and 452264 controls. A TWAS of AS was conducted using these data. The analysis software used was FUSION, and Epstein-Barr virus–transformed lymphocytes, transformed fibroblasts, peripheral blood, and whole blood were used as gene expression references. Gene ontology (GO) and Kyoto Encyclopedia of Genes and Genomes (KEGG) enrichment analyses were performed for the important genes identified *via* TWAS. Protein-protein interaction (PPI) network analysis based on the STRING database was also performed to detect genes shared by TWAS and mRNA expression profiles in AS. TWAS identified 920 genes (P <0.05) and analyzed mRNA expression profiles to obtain 1183 differential genes. Following comparison of the TWAS results and mRNA expression characteristics, we obtained 70 overlapping genes and performed GO and KEGG enrichment analyses of these genes to obtain 16 pathways. *Via* PPI network analysis, we obtained the protein interaction network and performed MCODE analysis to acquire the HUB genes. Similarly, we performed GO and KEGG analyses on the genes identified by TWAS, obtained 98 pathways after screening, and analyzed protein interactions *via* the PPI network. Through the integration of TWAS and mRNA expression analysis, genes related to AS and GO and KEGG terms were determined, providing new evidence and revealing the pathogenesis of AS. Our AS TWAS work identified novel genes associated with AS, as well as suggested potential tissues and pathways of action for these TWAS AS genes, providing a new direction for research into the pathogenesis of AS.

## Introduction

Ankylosing spondylitis (AS) is a chronic inflammatory disease that primarily affects the axial skeleton (1) and is a common rheumatic disease characterized by chronic lower back pain and spinal ankylosing (2). AS usually causes chronic back pain and morning stiffness in patients, resulting in restricted spinal cord mobility (3), which adversely affects the quality of life of patients. Studies have shown that AS is a prevalent and potentially incapacitating chronic inflammatory arthritis, affecting 0.5% to 1.5% of the western population (4).

With the development of genetics, increasingly more researchers are focusing on the genetic basis of AS. Genome-wide association studies (GWAS) are a powerful method to identify disease and trait-related variants of AS (5). Thus far, GWAS have determined that 113 single nucleotide polymorphisms (SNPs) affect the risk of AS, and ongoing GWAS research may identify more than 100 new risk sites (6). However, the reliability of GWAS in assessing the risk of complex diseases is limited as most SNPs recognized by GWAS are located in the non-coding region of the disease genome (7). Therefore, the resulting SNPs indisputably lack the explanatory power for the disease. To alleviate problems associated with statistical power and interpretability, a recent trend in large-scale association studies is the transcriptome-wide association study (TWAS) (8). Some studies have proposed that the whole TWAS that integrates GWAS and expression quantitative trait loci (eQTL) data are relatively effective at unraveling gene-trait associations (9). Recently, increasingly more researchers have used TWAS to identify susceptibility sites associated with complex diseases. For example, the study uses eQTL data for TWAS to improve the current largest-scale schizophrenia GWAS summary statistics (10). Recently, researchers performed a large TWAS of prostate cancer (PrCa), in which multiple associations between genetically predicted gene expression and PrCa risk were identified (11). A study using self-brain, blood, and adipose tissue as a gene expression reference for schizophrenia was performed, and following co-localization with mRNA expression profiles, 157 schizophrenia-associated genes were identified, 35 of which were novel (12).

In this study, for the first time, a large-scale AS GWAS data set was used to identify genetic sites that may be associated with AS by TWAS, and perform enrichment analysis and PPI network analysis on the identified genes to identify AS-related genes and biological pathways. Our research has discovered new genetic loci related to AS and provided more research directions for AS research.

## Materials and Methods

### GWAS Summary Data of AS

The GWAS summary data for AS were derived from UK Biobank samples (UK Biobank fields: 20002_1313). Briefly, the British Biobank gene data set contains the genome-wide genotype data of 452264 participants, including 1265 AS patients. DNA was extracted from stored blood samples of individuals, and genotyped using the UK Biobank Axiom array (13). There are 62,394 genotype variants that have passed quality control through the Applied Biosystems British Biobank Axiom Array. This data set contains 9,113,133 calculation variants after filtering (13). The IMPUTE4 program is used to perform responsibilities (http://jmarchini.org/software/). Detailed information regarding subjects, genotypes, blame, and quality control can be found in published studies (14). We retrieved the results of previous GWAS studies on ankylosing spondylitis presented in **Table 1**.

**Table 1:** Results of previous GWAS studies on ankylosing spondylitis.

First author	Year	People	SNP	Gene
Lin et al. (15)	2011	Asian	rs4552569	ANO6
			rs17095830	HAPLN1-EDIL3
			rs13210693	
Australo-Anglo-American Spondyloarthritis Consortium (TASC) et al. (16)	2010	European	rs11209026	IL23R
			rs4672495	
			rs10865331	
			rs2310173	IL1R2
			rs4333130	ANTXR2
			rs27037	ERAP1
			rs27434	ERAP1
			rs3734523	MHC
			rs2242944	
Evans et al. (17)	2011	European	rs11209026	IL23R
			rs2297909	KIF21B
			rs10865331	
			rs30187	ERAP1
			rs4349859	HLA-B
			rs378108	
			rs11249215	RUNX3
			rs6556416	IL12B
			rs11616188	LTBR-TNFRSF1A
			rs4389526	ANTXR2
			rs10440635	PTGER4
			rs10781500	CARD9
			rs8070463	TBKBP1
Li et al. (18)	2019	Turkish	rs10000518	HS3ST1
			rs34939008	GBA3
			rs10280089	FAM183B
			rs4876208	MYOM2
			rs1013210	ADAM28
			rs148783236	USP8
			rs11639037	TLE3
			rs8036083	CHSY1
			rs61752717	MEFV
			chr18:14723700:D	ANKRD30B
			rs235316	PTTG1IP
		Iranian	rs13001372	2p15
			rs27529	ERAP1
			rs7874251	PCSK5
			rs2031610	FAS
			rs7298011	RASSF8
			rs4808624	DDA1

### The TWAS of AS

In this study, FUSION software was used to analyze the GWAS aggregated data of TWAS to AS (http://gusevlab.org/projects/fusion/). FUSION is a set of tools used to evaluate the association between gene expression and target disease/phenotype based on pre-calculated gene expression weights and GWAS summary data (19). Briefly, this study used the predictive model in FUSION to calculate gene expression. Thereafter, the calculated tissue-related expression weight was combined with summary-level GWAS results, and transcriptional regulation was used as an intermediary between genetic variation and phenotype, and the association between a single genetic variation and phenotype was converted into genes/transcripts and phenotypes ‘S association. In previous studies, Epstein-Barr virus–transformed lymphocytes (EL), transformed fibroblasts (TF), peripheral blood (NBL), and whole blood (YBL) were used for rheumatoid arthritis (RA) TWAS analysis (20). In the pathogenesis of AS, various types of cells and tissues are attacked, including bones, cartilage, muscles, ligaments, synovium, fibroblasts, immune cells, etc. Therefore, for AS, which is also an autoimmune disease, the use of four gene expression weights were selected as a reference in this study. The gene expression weight panel for pre-calculation was downloaded from the FUSION website (http://gusevlab.org/projects/fusion/).

### Gene Expression Profiles of AS

We downloaded the mRNA expression profile of AS from the Gene Expression Gene Expression Omnibus DataSets (https://www.ncbi.nlm.nih.gov/geo/accession number: GSE11886). The platform of the GSE11886 chip is GPL570, Affymetrix Human Genome U133 Plus 2.0 Array expression beads, and tested samples are from eight AS patients (median course of disease 13 years [range <1–43 years]) and nine healthy control subjects 7 d old peripheral blood. We selected eight peripheral blood samples from AS patients and nine normal peripheral blood samples from the control group for differential gene analysis. The R language limma package was used for differential gene expression analysis. We screened genes with a folding change (FC) >2 or <0.5 (|log2FC |> 1) and an adjusted P value <0.05 as differentially expressed genes.

### Gene Enrichment Analysis

For genes identified using TWAS and mRNA expression profiles, Kyoto Encyclopedia of Genes and Genomes (KEGG) and gene ontology (GO) were used to identify and confirm related biological processes. The enrichment of KEGG and GO was done using R package’s “org.Hs.eg.db,” “clusterProfiler”.

### Protein-Protein Interaction Network Analysis

PPI network analysis was performed in this study using the STRING v11.5 database (STRING, https://string-db/org) to complete, the interactive network is generated based on the experiment with the required 0.15 confidence score and “active interactive source” (21).

## Results

### The TWAS Results of AS

**Figure 1:**
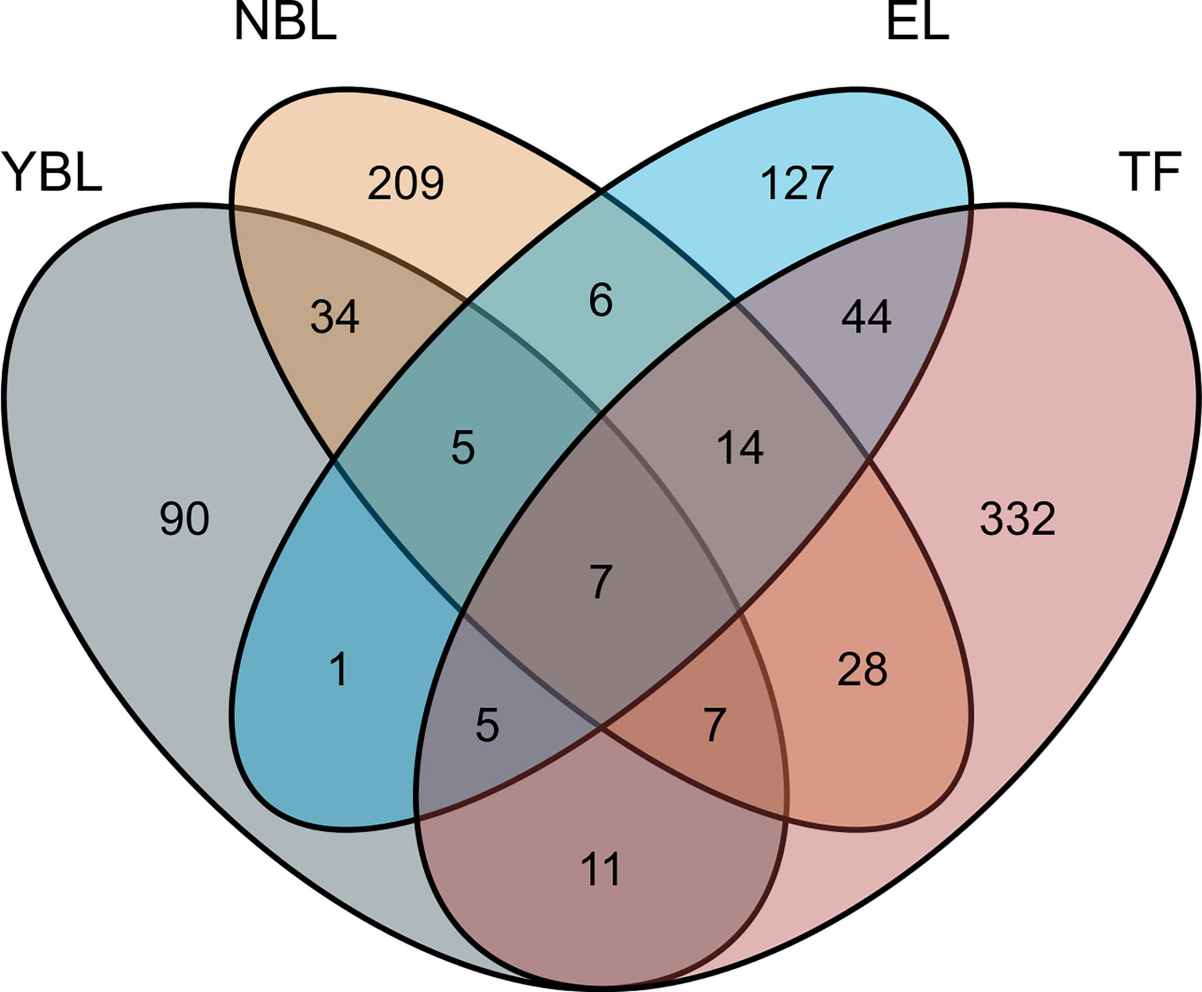
Venn diagram of genes obtained from TWAS identification in four: Gray, YBL; orange, NBL; blue, EL; pink, TF.

**Figure 2:**
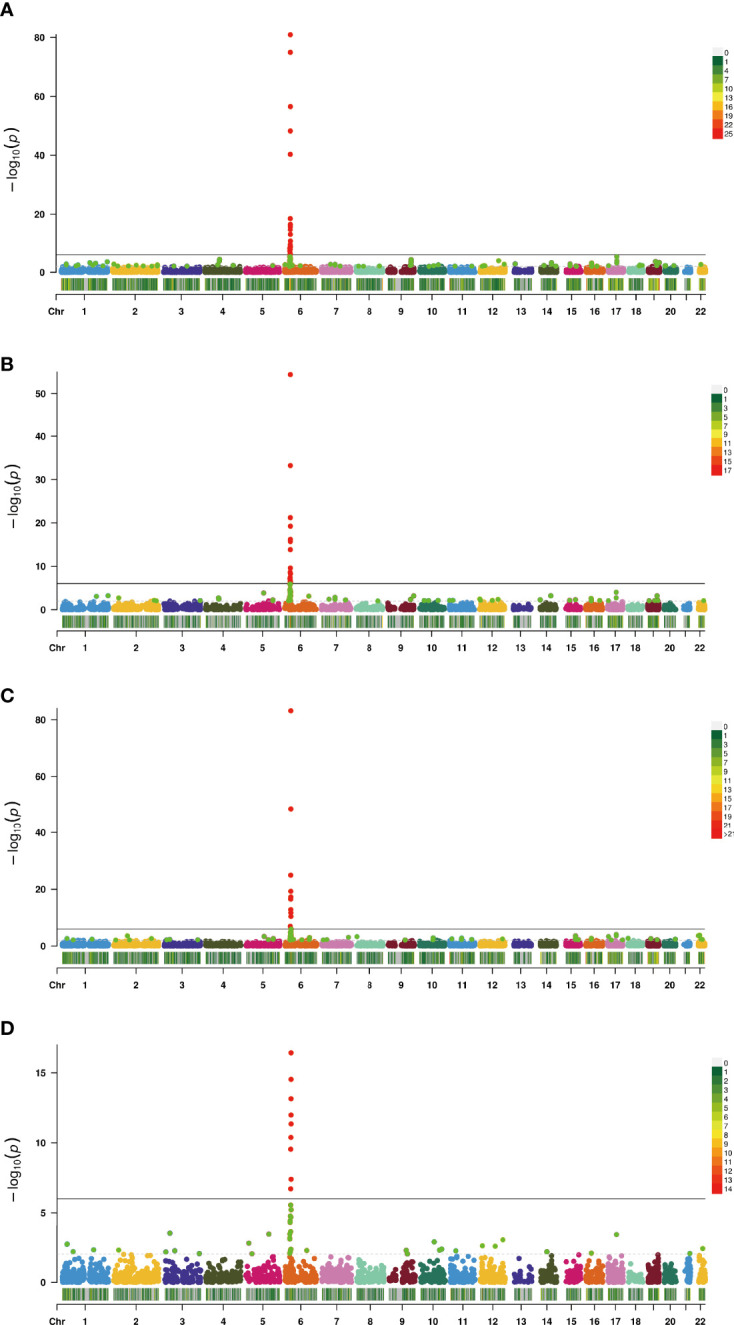
Manhattan plot of AS-associated genes identified by TWAS (colored dots). Each dot represents a gene, the x-axis is the physical location (chromosomal localization) and the y-axis is the -log10 (p-value) of the gene’s association with AS. Significant genes in different tissues/cells are highlighted in different colors (**A**, TF; **B**, EL; **C**, YBL; **D**, NBL).

In this study, the TWAS identified 920 genes, which included 448, 209, 159, and 310 genes of EL, TF, NBL, and YBL, respectively. All genes are shown in **Figure 1**. To find the specific and the most representative genes of each tissue, we performed overlap analysis on the genes identified by the TWAS in different tissues/cells. The overlap result is shown in the Venn diagram (**Figure 2**). For example, in TF, 332 genes were identified by the TWAS that are related to AS; there are 44 important genes in EL and TF; 14 in EL, TF, and YBL, and seven identified in the four joint identifications. The seven TWAS significant AS susceptibility genes jointly identified in the four tissues/cells are *LIPT1*, *LARS*, *MICA*, *MICB*, *BTN3A2*, *HLA-A*, and *ACTA2*. The detailed information of these seven overlapping genes are listed in **Table 2**, including the tissue/cell name, gene name (Gene), CHR and the most important GWAS Snp rsid (BEST.GWAS.ID), TWAS Z score (Zc), and TWAS P value (P).

**Table 2:** AS-significant expression genes for AS in all 4 tissues.

Tissue/cell	Gene	CHR	BEST.GWAS.ID	Z	P
TF	RNASEH2B	13	rs1927742	3.713471	0.000204
TF	BAG6	6	rs3819299	8.4484	2.95E-17
TF	FUCA1	1	rs2229579	3.703542	0.000213
TF	CYP21A1P	6	rs6929796	6.6759	2.46E-11
TF	VARS2	6	rs3819299	6.2585	3.89E-10
EL	ACTA2	10	rs3740286	4.412	1.02E-05
EL	ERAP1	5	rs27529	6.0093	1.86E-09
EL	TRIT1	6	rs3819299	9.15089	5.65E-20
EL	RP11-611D20.2	9	rs11145835	4.14	3.47E-05
EL	H6PD	6	rs3819299	6.33014	2.45E-10
NBL	USP3	15	rs11635675	4.004	6.23E-05
NBL	ZNF213	16	rs8047557	3.94091	8.12E-05
NBL	ACTA2	10	rs3740286	3.9118	9.16E-05
NBL	BTN2A1	6	rs7749823	4.123	3.73E-05
NBL	BTN3A1	6	rs7749823	3.563	3.67E-04
YBL	FASTKD5	20	rs2145222	3.799263	0.000145
YBL	CDC42SE1	1	rs4970991	3.71217	0.000205
YBL	GPT	8	rs748193	3.3464	0.000819
YBL	GTF2H4	6	rs2233972	8.4466	3.00E-17
YBL	HLA-C	6	rs2233972	7.3854	1.52E-13

CHR, chromosome; BEST.GWAS.ID rsID of the most significant GWAS SNP in locus, TWAS.Z, TWAS Z-score; P, TWAS P value.

### Common Genes Shared by the TWAS and mRNA Expression Profiling

We further compared genes recognized by the TWAS with the differential genes recognized by AS mRNA expression profile analysis. We detected 70 common genes shared by TWAS and mRNA expression analysis, such as *RETSAT*(P value=0.0243), *EOMES*(P value=0.00368), *CXCR6*(P value=0.0284), *PPIC*(P value=0.00496), and *TCF19*(P value=0.0481). The 10 genes commonly shared by the TWAS, and mRNA expression analysis are shown in **Table 3**.

**Table 3:** The common genes identified by both TWAS and microarray expression data.

Tissue/cell	Gene	CHR	BEST.GWAS.ID	P	Regulation
NBL	RETSAT	2	rs3923229	0.03451	up
NBL	EOMES	3	rs11129295	0.03302	down
NBL	CXCR6	3	rs1546076	0.03847	down
NBL	PPIC	5	rs7735164	0.000372	up
YBL	TCF19	6	rs2233972	2.40E-05	up
YBL	MICA	6	rs2233972	4.67E-05	up
YBL	DDAH2	6	rs2523586	5.92E-04	up
EL	C6orf48	6	rs3819299	1.14E-04	up
TF	DSN1	20	rs6130176	0.03601	up
TF	RTEL1	20	rs2252258	0.04847	up

CHR, chromosome; BEST.GWAS.ID rsID of the most significant GWAS SNP in locus.

### Gene Enrichment Analysis Results

**Figure 3:**
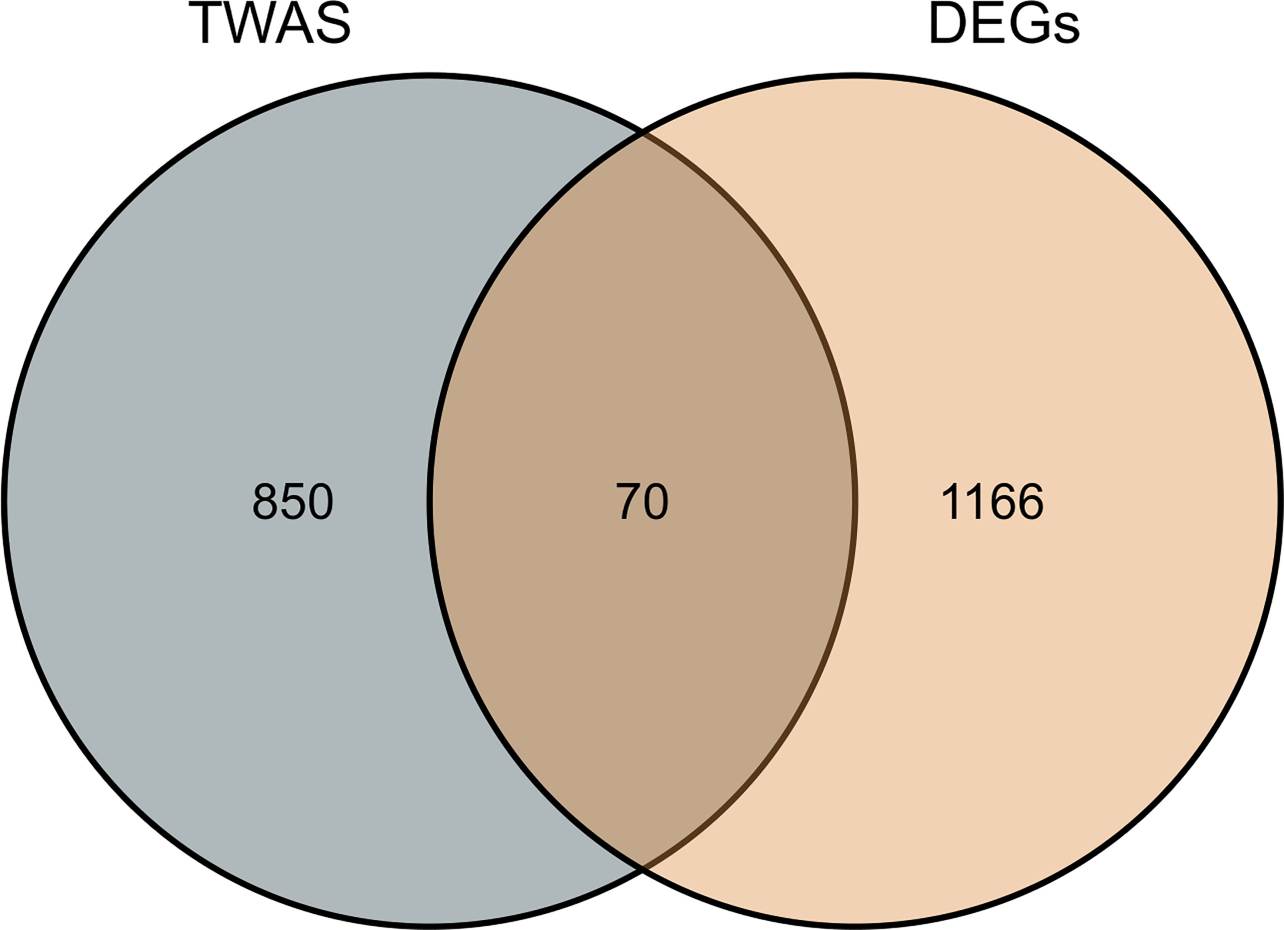
Venn diagram of TWAS versus common genes identified by mRNA expression profiling. Gray, TWAS; Orange, DEGs.

**Figure 4:**
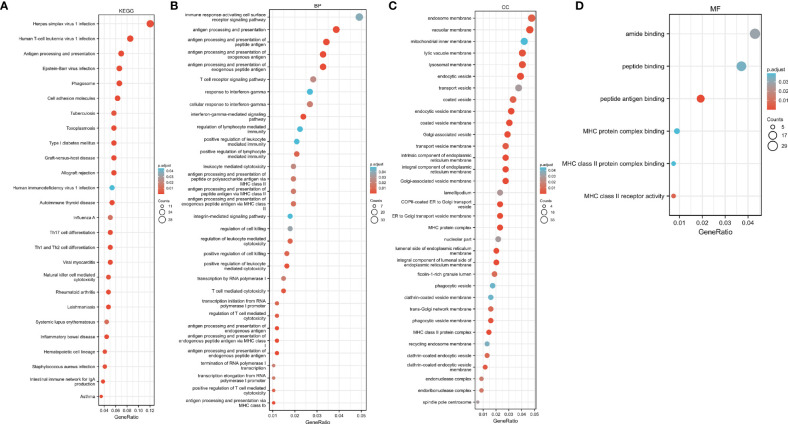
Network diagram of GO term analysis for TWAS-identified genes, where each circular point in the network represents a term whose size is proportional to the number of input genes for that term. **(A)** KEGG; **(B)** BP; **(C)** CC; **(D)** MF.

**Figure 5:**
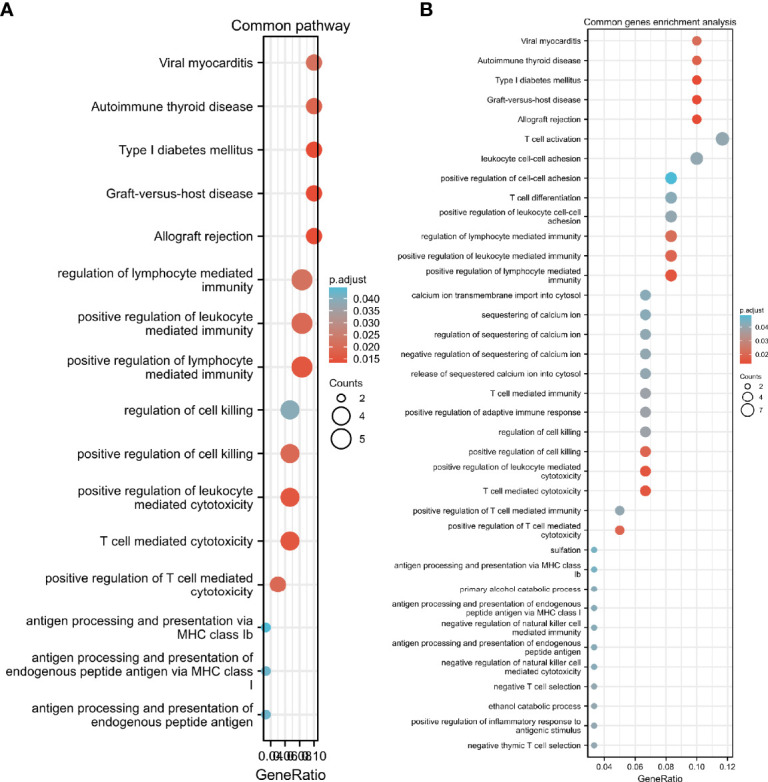
GO term analysis network diagram for TWAS-identified genes, where each circular dot in the network represents a term whose size is proportional to the number of input genes for that term. **(A)** Common pathway; **(B)** Common genes enrichment pathways.

We performed GO and KEGG pathway enrichment analysis on 920 genes identified using TWAS. Seventy-two GO terms, with p.adjust <0.05 and 26 KEGG terms with p.adjust <0.05, such as antigen processing and presentation were detected in the GO enrichment pathway. The results for endogenous peptide antigen (p.adjust = 3.31342E-05), interferon-γ-mediated signaling pathway (p.adjust = 0.000207229), antigen processing and presentation through MHC class Ib (p.adjust = 0.000363369), allograft rejection (p.adjust = 1.28509E-13), RA in the KEGG pathway (p.adjust = 5.07762E-05)), systemic lupus erythematosus (p.adjust = 0.010482717), etc. are shown in **Figure 3**. Similarly, we also performed GO and KEGG pathway enrichment analysis on common genes identified by the TWAS in the mRNA expression profile. Thirty-two GO terms with p.adjust <0.05 and 5 KEGG terms with p.adjust <0.05 were detected. The results for T cell–mediated cytotoxicity (p.adjust = 0.015712930095128), leukocyte-mediated positive immune regulation (p.adjust = 0.019575169), etc. are shown in **Figure 4**. Further comparison of TWAS and common gene enrichment analysis results detected 16 common pathways with p.adjust <0.05, such as antigen processing and presentation of endogenous peptide antigens, MHC class I antigen processing and presentation of endogenous peptide antigens, etc. (**Figure 5**).

### PPI Network Analysis

**Figure 6:**
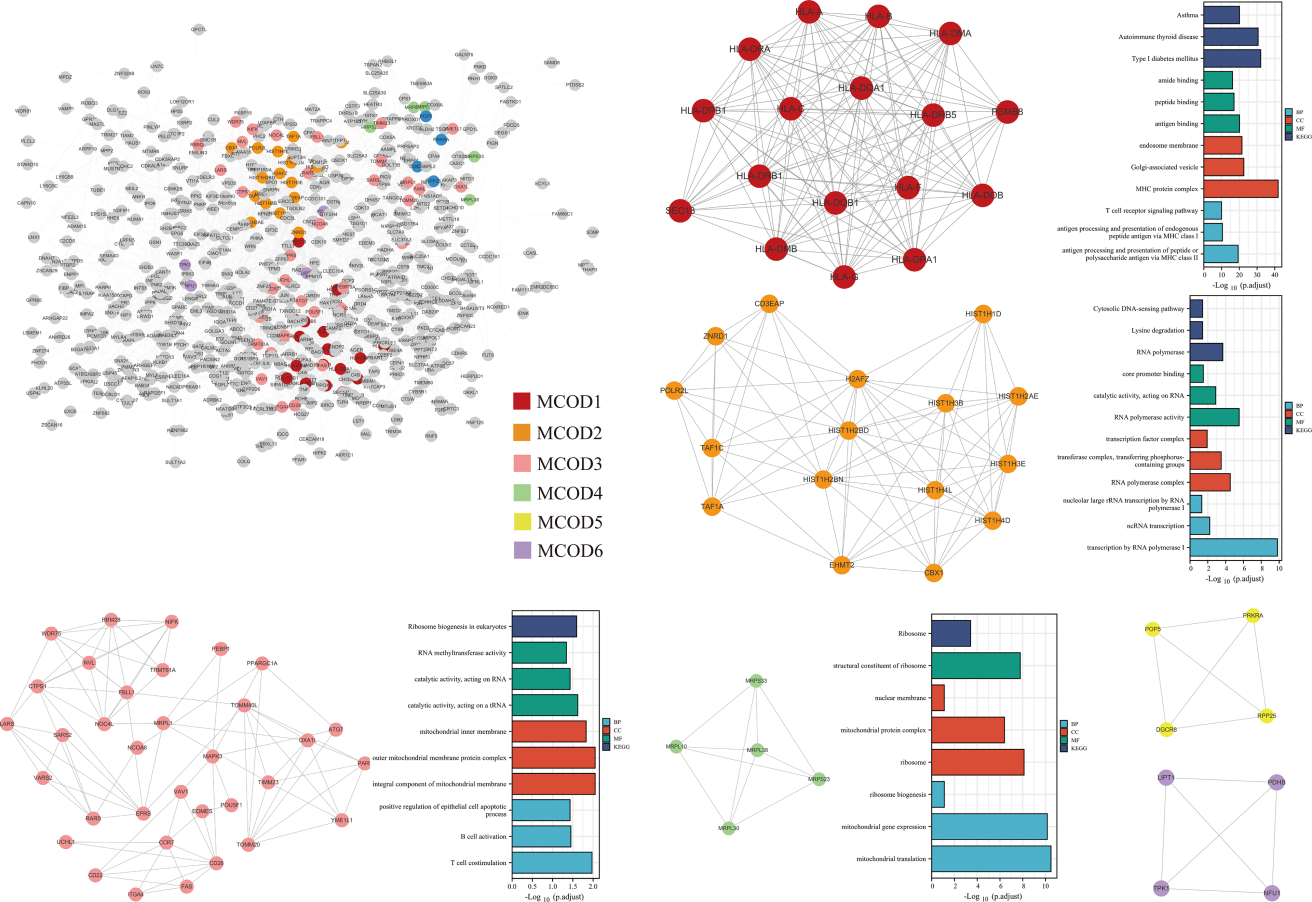
PPI network of the TWAS-identified genes and their functional exploration.

The PPI network was constructed based on 920 genes identified by the TWAS. We further analyzed the constructed PPI network using MCODE. The important modules from the PPI network formed six MCODE groups with a class of genes (**Figure 6**).

## Discussion

Recently, TWAS has been proposed as a principled approach to integrate eQTL analyses with GWAS to identify genes whose genetically regulated expression is associated to disease risk (22). The TWAS has garnered substantial interest and become increasingly popular within the human genetics research community (23). Effect sizes of cis-eQTLs were utilized to impute gene expression from genotyped samples in the TWAS (24). It captures heterogeneous signals better than individual SNPs or cis-eQTLs and focuses prediction on the genetic component of expression that avoids confounding factors from environmental differences caused by the trait that may influence expression (20). In this study, the TWAS is the first in the field of AS susceptibility gene research.

Ankylosing spondylitis (AS) is a chronic inflammatory disease of unknown etiology and, unlike other systemic autoimmune diseases, the innate immune system has a dominant role in AS (25). It is an important chronic inflammatory disease with an incidence between 0.5% and 1.0% (26). In AS, inflammation most likely originates at the tendon–bone interface (an example of enthesitis), leading to erosion and bone proliferation *via* mechanisms that are poorly understood (27). AS is a common chronic immune mediator disease characterized by inflammation of the axial bones, and there are extra-articular manifestations such as the co-occurrence of psoriasis and inflammatory bowel disease, with uveitis being the most common (27). In the pathogenesis of AS, various types of cells and tissues are attacked, including bones, cartilage, muscles, ligaments, synovium, fibroblasts, immune cells, etc. The greatest contributor to AS is HLA-B27 (Human leukocyte antigen); more than 90% of AS patients are HLA-B27 positive, thus the HLA-B27 factor in the blood is primarily used to diagnose AS (28). Therefore, in this study, we used four tissues/cells as a reference for gene expression, and conducted a TWAS on AS.

Among the genes identified by the TWAS, together with mRNA expression profiles, we found multiple susceptibility genes for AS, which can be studied further. Eomesodermin (EOMES) are T-box transcription factors that drive differentiation and function of cytotoxic lymphocytes, such as NK cells (29). It has been shown that although several transcription factors are important for NK cell maturation and function, T-box-related T-BET (encoded by TBX21) and EOMES transcription factors are essential for terminal NK cell maturation and function as mice lacking Tbx21 and EOMES lack mature NK cells (30). The chemokine receptor, CXCR6, is an important receptor for CXCL16 (31). It has been shown that Fc fusion proteins reduce the RANCL/OPG ratio by inhibiting the CXCL16/CXCR6 pathway, thereby suppressing inflammation and bone destruction in AS (32). Heme 4D (Sema4D) is expressed on T cells and osteoblasts and regulates T cell proliferation and skeletal remodeling, and findings suggest that Sema4D, a potent activator of T cells in the immune response, contributes to inflammation in AS by inducing imbalances in Th17 and Treg cell populations in an AhR-dependent manner, indicating that it is a key player in the pathogenesis of AS (33).

In this study, we used four tissues/cells as a reference for gene expression and conducted a TWAS on AS. We used the TWAS to identify 920 statistically significant genes in TF, EL, YBL, and NBL. Among them, seven genes *LIPT1*, *LARS*, *MICA*, *MICB*, *BTN3A2*, *HLA-A*, and *ACTA2* were jointly identified using the four tissues/cells. Seventy genes were jointly identified using the TWAS and mRNA expression profiles, among which *MICA* and *HLA-A* were also identified using the four tissues/cells.

HLA-B27 and AS are a paradigmatic example of association between the HLA and an immuno-mediated disease (34). HLA molecules are highly polymorphic, HLA -A, HLA -B, and HLA -DRB1 belong to the same family of molecules and are considered closely related to allogeneic rejection and autoimmune reactions (35). MICA interacts with NKG2D on immune cells to regulate the host immune response, which is highly related to the autoimmune response (36). MICA is a member of the non-classical MHC class I family and is highly polymorphic. The promoter SNP rs4349859 of MICA, previously used as an HLA-B27 tag SNP, has the strongest association with European AS (37). This evidence supports that the application of the TWAS in AS is relatively effective. It can be seen that the genes identified in this study can elucidate the mechanism of AS.

Studies using one or more anti-rheumatic drugs (methometamine, hydroxychloroquine, or sulfonamide) on 46 patients with suspected RA, followed by a genome-wide methylation analysis of baseline T lymphocyte DNA found that the *BTN3A2* gene is Two CpG sites are closely related to a treatment response (38). AS and RA are both autoimmune diseases; the CpG locus of the *BTN3A2* gene may be related to AS, which provides a new research direction for the pathogenesis of AS. Some studies have shown that three human mitochondrial diseases that directly affect lipoic acid metabolism result from heterozygous missense and nonsense mutations in *LIAS*, *LIPT1*, and *LIPT2* genes (39). It has been shown that the oxidative serum environment of AS promotes mesenchymal stem cell aging by inducing mitochondrial dysfunction and excessive ROS production (40), which suggests that the pathogenesis of AS is closely related to mitochondrial dysfunction, further suggesting that LIPT1 may be related to the pathogenesis of AS. Moreover, aberrant regulation of autophagy has been implicated in an increasing number of autoimmune disorders such as systemic lupus erythematous, RA, AS, multiple sclerosis, Crohn’s disease, and vitiligo (41). It has been shown that autophagy in fibroblasts correlates with mRNA levels of ACTA2, TGF-1, FGF2, and PDGFRA (42). This suggests that ACTA2 may cause AS *via* induction of fibroblast autophagy.

GO enrichment analysis detected several GO terms such as T cell–mediated cytotoxicity, positive regulation of T cell–mediated cytotoxicity, lymphocyte mediated immune regulation, cell killing regulation, Th17 cell differentiation, antigen processing and presentation *via* MHC class I for endogenous peptide antigens, antigen processing and presentation *via* MHC class Ib, autoimmune thyroid disease, etc. Some researchers have detected cd4t cells with cytotoxic potential in RA and AS and are able to mediate graft rejection through a perforin-dependent mechanism (43). It has also been suggested that local HLA-dependent activation of peptide-specific cytotoxic CD8+ T cells in chondrocytes may play a role in the inflammatory process of AS (44). It has been suggested that genes associated with AS (*HLA-B27*, *ERAP-1*, and *KIR*) may affect NK cell function. There are some functional changes in a subset of NK cells that suggest an NK cell response similar to the Th1 pathway, and some changes in NK cell phenotype/function can be used to predict treatment response in patients with AS (45). IL-17 is a cytokine implicated in the pathophysiology of AS, and researchers have used anti-IL-17 monoclonal antibodies for the treatment of AS and found significant efficacy (46). AS is closely associated with the MHC class I allele HLA-B27, which strongly suggests that our use of the TWAS to identify the resulting gene is highly effective (47). These identified biological pathways have been shown to have some association with AS, providing us with additional areas of research interest.

In this study, we used TWAS and mRNA expression profiling together to identify susceptibility genes for AS. The TWAS can detect genes associated with AS at the DNA level, mRNA expression profiling can explain the pathogenesis of AS at the protein expression level, and the combination of the two can more accurately identify susceptibility genes for AS. Our research has identified new genes and biological pathways associated with AS, providing additional research directions for the study of AS.

However, the study has some limitations. First, the GWAS pooled data are from the UK Biobank, and study subjects are predominantly from European populations. Therefore, the results of this study should be used with caution when studying AS in other populations. Second, some susceptibility genes of AS obtained from the analysis have not been verified *via* molecular biology experiments, which should be performed in future studies.

## Data Availability Statement

The datasets presented in this study can be found in online repositories. The names of the repository/repositories and accession number(s) can be found in the article/**Supplementary Material**.

## Author Contributions

RF, ML and PX designed the study. All authors contributed to the article and approved the submitted version.

## Funding

This work was supported by the National Natural Scientific Foundation of China (82072432, 81772410).

## Conflict of Interest

The authors declare that the research was conducted in the absence of any commercial or financial relationships that could be construed as a potential conflict of interest.

## Publisher’s Note

All claims expressed in this article are solely those of the authors and do not necessarily represent those of their affiliated organizations, or those of the publisher, the editors and the reviewers. Any product that may be evaluated in this article, or claim that may be made by its manufacturer, is not guaranteed or endorsed by the publisher.

## Supplementary Material

Supplementary Table 1 TWAS result of AS.

Supplementary Table 2 AS TWAS detects candidate genes GO and KEGG enrichment analysis results.

Supplementary Table 3 The intersection result of the pathway enrichment analysis result of the common gene and the AS TWAS enrichment pathway.

Supplementary Table 4 Common genes.

## Abbreviations

AS, Ankylosing spondylitis; RA, Rheumatoid arthritis; EL, EBV-transformed lymphocytes; TF, transformed fibroblasts; NBL, peripheral blood; YBL, whole blood; GWAS, Genome-wide association study; TWAS, Transcriptome-wide association studies; eQTL, expression quantitative trait Loci; DEGs, Differentially expressed genes; GTEx, Genotype-Tissue Expression; PPI, protein-protein interaction; GO, Gene ontology; KEGG, Kyoto Encyclopedia of Genes and Genomes; HLA, Human leukocyte antigen; MHC, Major histocompatibility complex; MICA, Major Histocompatibility Complex Type I Chain Related Gene A.
